# Supplementary material for: Innovative data techniques for centrifugal pump optimization with machine learning and AI model
Source: PLoS One. 2025 Jun 10;20(6):e0325952. doi: 10.1371/journal.pone.0325952 (PMC12151477; doi:10.1371/journal.pone.0325952)
Supplement: Attachment — Submitted filename: Review.pdf [file pone.0325952.s001.pdf]

The article presents a machine learning (ML) and deep learning (DL) framework for predictive maintenance of centrifugal pump machines (CPM), focusing on automating condition monitoring and fault detection. The authors highlight the limitations of traditional monitoring methods, such as manual data analysis, which is time-consuming and lacks adaptability across various operating conditions. By implementing a five-point summary of the data, exploratory data analysis (EDA), feature engineering, and data visualization, the study seeks to enhance data quality before model training. The ML models used, including random forest and deep learning classifiers, are evaluated based on metrics such as accuracy and F1-score, achieving high precision in detecting pump conditions. The proposed approach demonstrates an efficient, robust alternative for condition monitoring in industrial applications, with the potential for broad adaptation in predictive maintenance workflows.

Below are my recommendations for each section of the article.

Abstract:

- The abstract has long sentences that make it difficult to read and understand the objectives and results of the study.
  - Simplify the writing to highlight objectives and results in an objective manner
- There is no quantitative presentation of the main results
  - Include a sentence containing quantitative results, such as accuracy or F1-score, to provide a clear view of the model's effectiveness.
- Sentence: ***“The Dewsoft FFT DAQ system is designed to extract the high-quality data from the CPM based on sensor fusion technology.”***
  - Check the correct name of the system (probably "Dewesoft") and use it consistently throughout the article
- Sentence: ***“… to transform raw data into a valuable resource that will be adapted by ML & AI Models.”***
  - Inappropriate use of the verb adapted. Replace with “… to transform raw data into a valuable resource that can be utilized by ML and AI models.”

Introduction:

- Include a discussion of gaps in existing approaches to motivate the need for the study.
  - The sentence ***"However, existing methods often require complex feature engineering and are not universally applicable across varying operational conditions."*** → touches lightly on the limitations of existing methodologies but is insufficient to address literature gaps critically. For a complete analysis, the introduction needs to: 1) Specify concrete limitations of each work and compare them with the proposed approach.
- In the following sentence: ***“Exploratory data analysis (EDA) is typically conducted prior to modeling to ascertain informative features and discern patterns within the dataset.”***
  - There is a formatting issue in the lines
- In the following sentence, there is an inappropriate use of the word "improvise"

    - ***"K-means clustering was used by Antonio L. Alfeo et al. [6] to improvise the interpretation of model by achieving the best quality feature combination for a specific classification problem."***→ Replace "improvise" with "improve"
- In the following sentence, the subject is missing
    - ***"By applying advanced data analysis and machine learning to centrifugal pump data, strives to create reliable models that can detect flow obstructions..."***
        - Add the subject: "This study seeks to create models..."

Methodology:

- Improve the relationship between the text and the figures to improve the understanding of the methodology. Examples:
    - Sentence: ***"Figure 1 depicts the hardware and software that make up the model's architecture. Hardware includes CPM & DEWSOFT FFT DAQ system while Software includes Data Preprocessing, Data Visualization, Hypothesis Testing, Train AI & ML models, pickle, deploy final model, Fault Prediction and alerting system"***. → In Figure 1, it is not possible to visualize the terms DEWSOFT FFT DAQ system, and we do not visualize Data Visualization, Hypothesis Testing, Train AI & ML models, pickle, deploy final model, Fault Prediction, and alerting system.
    - Sentence: ***"The recorded data is further prepared, analyzed and visualized using Exploratory Data Analysis (EDA) before data transformation as observed in figure 2."*** → The sentence is too brief to explain the content of Figure 2.
- Use flowcharts to illustrate the methodology process more clearly and visually.
    - For example, adjust and relate the subsections of the methodology (including the names of the subsections) with a figure that represents the methodological process of the proposal.
- Figure 2 presents a block called EDA & FE. However, the acronym FE does not appear in the text.
- There is an inconsistency in the numbers presented about the data set
    - ***"...comprises 12 independent variables and 4 dependent variables, resulting in a dataset with 13 columns..."***
    - In Figure 4, we observe 11 variables.
    - Where are the 13 columns? Make this information clearer in the text
- Table 4
    - Define in text what each of the variables presented in Figure 4 are (what they are for, what they measure). Ex. Casing, C_temp, ...
    - Figure 4 should be transformed into a Table
- Below, there is colloquial language
    - ***"Data is a fuel for machine learning model which plays a vital role."***
        - Rewrite to a more academic tone
- The list of data cleaning steps is incomplete.
    - ***"Primary steps for data cleaning consist of: Eliminating Null data; Transforming Categorical data; Dealing with noise & outliers"***
    - The step of removing duplicate data is missing

- There is a grammatical error in the following sentence.
    - ***"However, the collected data may often contain noise, outliers, or missing values, it may impair the analysis's accuracy."***
    - Replace the verb impair with compromise
- The following sentence is confusing in the description of EDA.
    - ***"Exploratory Data Analysis (EDA) contains the methodology that includes statistical investigation..."***
    - Suggestion: Exploratory Data Analysis (EDA) includes methodologies such as statistical investigation, treatment of null and duplicate values, and identification of outliers...
- The following sentence is poorly worded
    - ***"Applying EDA is basic & essential process which considers 70% of work completion."***
    - Suggestion: Performing EDA is fundamental and comprises approximately 70% of the total effort in the analysis process.
- The following explanation about when to use the Z and T-tests seems incorrect.
    - ***"When the sample size is greater than or equal to 30, the Z-test is employed. T-Test uses unpaired and paired approach but the sample size should be below 30."***
    - Suggestion: The Z-test is used when the population standard deviation is known, or the sample size is large ($n \geq 30$). The T-test is used when the population standard deviation is unknown, and the sample size is small ($n < 30$).
- The following sentence is not precise about the use of tests in relation to standard deviation
    - ***"Also, if the SD of data is known then it is preferred to use Z-test if it is not known then use T-test but still the sample size should be less than 30."***
    - Suggestion: If the population standard deviation is known, use the Z-test regardless of the sample size. If it is unknown and the sample is small, use the T-test.
- There is a lack of clarity in the following sentence
    - ***"Z-test has failed to reject the null hypothesis... Anova test has also fail to reject Null Hypothesis..."***
    - Suggestion: The Z-test failed to reject the null hypothesis, indicating no significant difference between the means of the training and test sets for the "casing" feature. Likewise, the ANOVA test failed to reject the null hypothesis, suggesting that the means of the original, training, and test sets are statistically equal.
- The caption for Figure 5 has some typographical issues
    - ***"Figure5:HypothesisTestingofCPMdataset"***
- The hypothesis testing section uses complex language that can be simplified.
    - ***"The p-value has to be determined for sample data on the basis of that null and alternate hypothesis test will give the outcomes for z-test & anova test. So, applying hypothesis for stats comparison of CPM data before and after preprocessing. The z-test is used to determine if there is a significant difference in the means of the features, whereas ANOVA is applied to check the variance across multiple groups, allowing us to ensure data consistency and quality."***

        - Simplify the language and explanation of the tests, focusing on the main conclusions.

Result and discussion

- A superficial description of the univariate analyses was provided.
    - ***"Univariate analysis, which examines one variable at a time, has been employed..."***
        - Provide specific details of the analyses performed and their results. This may help explain why there are 03 different graphs for the same variables, as discussed below.
- In the subsections Histogram Plot and Violin Analysis, Box Plot analysis
    - There is no mention of which variable is being measured, the frequency. For example, is the rotation or vibration of the Casing, Impeller, and Bearing being measured?
    - Why use 03 different graphs to present the same variable? I suggest choosing and keeping either a histogram, violin, or box plot.
- The following sentence is poorly formulated about Naive Bayes
    - "It's commonly used for Multi Classification."
    - Correct to: "It's commonly used for multiclass classification problems."

Conclusion:

- Inadequate insertion of a table in conclusion.
    - Table 4 Performance Table
    - Should be in the results and discussion section
    - The caption of the Table should be more specific about the performance of what is being presented on the table. Change the name of the caption to be more complete about what the table presents.
    - There is no citation of Table 4 in the text
- Contradiction about the performance of the RF model.
    - ***"However, the Random Forest classifier showed tendencies to overfit..."***
    - Explain in the results section, justifying the statement about Random Forest overfitting.

For all sections:

- Check the quality of the Figures
    - For example, violin plots and box plots have low resolution and are difficult to interpret.
    - Improve the resolution of the figures by exporting them to high-quality formats (for example, PNG with 300 dpi).
    - Standardize the style of the graphs, using axes with captions and clearly indicated units
