# Supplementary material for: Innovative data techniques for centrifugal pump optimization with machine learning and AI model
Source: PLoS One. 2025 Jun 10;20(6):e0325952. doi: 10.1371/journal.pone.0325952 (PMC12151477; doi:10.1371/journal.pone.0325952)
Supplement: Attachment — Submitted filename: Response to Reviewers.docx [file pone.0325952.s003.docx]

**Response to reviewers’ comments**

**Journal Requirements:**

**When submitting your revision, we need you to address these additional requirements.**

Reviewer #1: Please ensure that your manuscript meets PLOS ONE's style requirements, including those for file naming. The PLOS ONE style templates can be found at

<https://journals.plos.org/plosone/s/file?id=wjVg/PLOSOne_formatting_sample_main_body.pdf>

and

[https://journals.plos.org/plosone/s/file?id=ba62/PLOSOne_formatting_sample_title_authors_affiliations.pdf](https://track.editorialmanager.com/CL0/https:%2F%2Fjournals.plos.org%2Fplosone%2Fs%2Ffile%3Fid=ba62%2FPLOSOne_formatting_sample_title_authors_affiliations.pdf/1/010f019444a08961-667ddd56-8337-4b21-99a5-41e4e68259e6-000000/kYTmHsoAxz8KHD4--E7wtT9gvB550m5ONr12rnNKmgA=193)

>> The correction has been incorporated for heading, figures and tables styling in the revised manuscript as per the reviewer suggestion.

Please note that PLOS ONE has specific guidelines on code sharing for submissions in which author-generated code underpins the findings in the manuscript. In these cases, we expect all author-generated code to be made available without restrictions upon publication of the work. Please review our guidelines at <https://journals.plos.org/plosone/s/materials-and-software-sharing#loc-sharing-code> and ensure that your code is shared in a way that follows best practice and facilitates reproducibility and reuse.

>> The Sample code and data will be available without any restriction and also have disclosed the same in data availability statement before References in the manuscript.

PLOS requires an ORCID iD for the corresponding author in Editorial Manager on papers submitted after December 6th, 2016. Please ensure that you have an ORCID iD and that it is validated in Editorial Manager. To do this, go to ‘Update my Information’ (in the upper left-hand corner of the main menu), and click on the Fetch/Validate link next to the ORCID field. This will take you to the ORCID site and allow you to create a new iD or authenticate a pre-existing iD in Editorial Manager.

>> OrciD id has been authenticated and added in the Editorial Manager.

We note you have included a table to which you do not refer in the text of your manuscript. Please ensure that you refer to Table 4 in your text; if accepted, production will need this reference to link the reader to the Table.

>> The Correction of referring Table 4 has been incorporated in the revised manuscript as per the reviewer suggestion.

**Review Comments to the Author**

**Reviewer #1: The article presents a machine learning (ML) and deep learning (DL) framework for predictive maintenance of centrifugal pump machines (CPM), focusing on automating condition monitoring and fault detection. The authors highlight the limitations of traditional monitoring methods, such as manual data analysis, which is time-consuming and lacks adaptability across various operating conditions. By implementing a five-point summary of the data, exploratory data analysis (EDA), feature engineering, and data visualization, the study seeks to enhance data quality before model training. The ML models used, including random forest and deep learning classifiers, are evaluated based on metrics such as accuracy and F1-score, achieving high precision in detecting pump conditions. The proposed approach demonstrates an efficient, robust alternative for condition monitoring in industrial applications, with the potential for broad adaptation in predictive maintenance workflows.**

The Conclusion section has been reorganized and elaborated for easily understanding to readers. The Future work has been incorporated in the conclusion section too.

Attached are my recommendations for the article.

Reviewer #2: (1) The introduction discusses general challenges in centrifugal pump optimization but can be improved by explicitly stating the unique contributions of this study. For instance, clearly highlight how the Dewsoft FFT DAQ system and the applied machine learning techniques improve upon existing methods in terms of predictive accuracy and operational efficiency.

>> The Correction has been incorporated in the introduction section for Dewesoft FFQ System and highlighted as per the reviewer’s suggestion.

(2) The section on hypothesis testing (page 14, Figure 5) is well-structured but could benefit from a brief comparison of the results obtained through Z-tests and ANOVA for different preprocessing scenarios. Explain how these tests validate the absence of bias and their implications for the robustness of the machine learning models.

>> The Correction has been incorporated & highlighted in the hypothesis section for z-test and anova test also the figure number has been changed due to restructuration based on reviewers’ comment.

(3) Some figures, such as the heatmap on page 20 (Figure 12), are informative but lack sufficient descriptions to guide readers unfamiliar with correlation matrices. Add a legend or a few explanatory sentences to clarify key observations, such as which correlations are critical for predictive maintenance.

>> As per reviewers’ suggestion detailed observation and insight has been added in heatmap section based on co-relation.


(4) While the paper presents model performance (Tables 1–4, pages 24–29), the discussion should include a comparison of computational efficiency or training times across models like Naïve Bayes, SVC, and Random Forest. This would provide practical insights into model selection for real-time deployment.

>> The computational efficiency of ML & ANN models has been added in table and graph in of Results and Discussion section as per reviewers’ suggestion.


(5) The manuscript effectively discusses data preprocessing and modeling but should connect these findings to practical scenarios. For example, explain how the insights from clustering (e.g., Figures 13 and 14 on page 22) could inform maintenance schedules or reduce downtime in industrial settings.

>> The correction has been incorporated and highlighted in the revised manuscript as per the reviewer suggestion with linking the heatmap, scatter cluster and kmeans clustering to downtime maintenance schedule.

(6) I think the paper could benefit from the following papers that greatly investigated the optimal ML workflow:

<https://doi.org/10.15530/urtec-2024-4044244>
<https://doi.org/10.1016/j.geothermics.2024.103028>
<https://doi.org/10.2118/219231-MS>
<https://doi.org/10.3390/en15238835>

>> The paper has been beneficial for understanding the optimal ML/ ANN workflow and cited as per reviewers’ suggestion.

Below were the recommendation by reviewer:

Abstract:

- The abstract has long sentences that make it difficult to read and understand the objectives and results of the study.
  - Simplify the writing to highlight objectives and results in an objective manner

>> The suggested changes by reviewer in abstract section has been incorporated and highlighted.

- There is no quantitative presentation of the main results
  - Include a sentence containing quantitative results, such as accuracy or F1-score, to provide a clear view of the model's effectiveness.

>> The quantitative sentence such as F1-score, Precision and recall has been added and highlighted in the abstract section

# Sentence: “The Dewsoft FFT DAQ system is designed to extract the high-quality data from the CPM based on sensor fusion technology.”

- Check the correct name of the system (probably "Dewesoft") and use it consistently throughout the article

>> As per reviewers’ suggestion the correction in the name Dewesoft has been incorporated in whole manuscript.

# Sentence: “… to transform raw data into a valuable resource that will be adapted by ML & AI Models.”

- Inappropriate use of the verb adapted. Replace with “… to transform raw data into a valuable resource that can be utilized by ML and AI models.”

>> As per suggestion by reviewer the correction has been incorporated & highlighted in the abstract section.

Introduction:

- Include a discussion of gaps in existing approaches to motivate the need for the study.
  - The sentence ***"However, existing methods often require complex feature engineering and are not universally applicable across varying operational conditions."*** 🡪 touches lightly on the limitations of existing methodologies but is insufficient to address literature gaps critically. For a complete analysis, the introduction needs to: 1) Specify concrete limitations of each work and compare them with the proposed approach.

>> The limitation of existing methodologies and recent work has been added & highlighted in the second last paragraph of introduction as suggested by the reviewer.

# In the following sentence: “Exploratory data analysis (EDA) is typically conducted prior to modeling to ascertain informative features and discern patterns within the dataset.”

- There is a formatting issue in the lines

>> The formatting issue has been solved & highlighted as suggested by the reviewer.

- In the following sentence, there is an inappropriate use of the word "improvise"

# "K-means clustering was used by Antonio L. Alfeo et al. [6] to improvise the interpretation of model by achieving the best quality feature combination for a specific classification problem."🡪 Replace "improvise" with "improve"

>> The word “improvise” has been replaced with “improve” as suggested by reviewer.

- In the following sentence, the subject is missing

# "By applying advanced data analysis and machine learning to centrifugal pump data, strives to create reliable models that can detect flow obstructions..."

- Add the subject: “This study seeks to create models...”

>> As suggested by reviewer the subject “The study seeks to create models” has been incorporated and highlighted in the last paragraph of introduction section.

Methodology:

- Improve the relationship between the text and the figures to improve the understanding of the methodology. Examples:
  - Sentence: ***“Figure 1 depicts the hardware and software that make up the model's architecture. Hardware includes CPM & DEWSOFT FFT DAQ system while Software includes Data Preprocessing, Data Visualization, Hypothesis Testing, Train AI & ML models, pickle, deploy final model, Fault Prediction and alerting system”***. 🡪 In Figure 1, it is not possible to visualize the terms DEWSOFT FFT DAQ system, and we do not visualize Data Visualization, Hypothesis Testing, Train AI & ML models, pickle, deploy final model, Fault Prediction, and alerting system.

>> As suggested by reviewer the entire sentence has been structured and changes has been incorporated.

- Sentence: ***“The recorded data is further prepared, analyzed and visualized using Exploratory Data Analysis (EDA) before data transformation as observed in figure 2.”*** 🡪 The sentence is too brief to explain the content of Figure 2.

>> The correction has been incorporated & highlighted in the revised manuscript as per the reviewer suggestion.

- Use flowcharts to illustrate the methodology process more clearly and visually.
  - For example, adjust and relate the subsections of the methodology (including the names of the subsections) with a figure that represents the methodological process of the proposal.

>> As suggested by reviewer the changes have been incorporated and highlighted - The figure 2 highlights the general methodology while figure 3 has sub section with detailed explanation of the methodology.

- Figure 2 presents a block called EDA & FE. However, the acronym FE does not appear in the text.

>> The correction for FE (Feature Engineering) has been incorporated & highlighted in the revised manuscript as per the reviewer suggestion.

- There is an inconsistency in the numbers presented about the data set

# "...comprises 12 independent variables and 4 dependent variables, resulting in a dataset with 13 columns..."

- In Figure 4, we observe 11 variables.
- Where are the 13 columns? Make this information clearer in the text
- Table 4
  - Define in text what each of the variables presented in Figure 4 are (what they are for, what they measure). Ex. Casing, C_temp, .....
  - Figure 4 should be transformed into a Table

>> As suggested by reviewer the figure 4 has been converted to Table 1. Also, the clarity between name and no of variables/ features displayed in 5-point summary vs data has been explained and highlighted in the section.

- Below, there is colloquial language

# "Data is a fuel for machine learning model which plays a vital role."

- Rewrite to a more academic tone

>> The correction has been incorporated and highlighted in the revised manuscript as per the reviewer suggestion.

- The list of data cleaning steps is incomplete.

# "Primary steps for data cleaning consist of: Eliminating Null data; Transforming Categorical data; Dealing with noise & outliers"

- The step of removing duplicate data is missing

>> As per the reviewer suggestion the “duplicate data” has been added and highlighted in Data analysis & pre-processing section.

- There is a grammatical error in the following sentence.

# "However, the collected data may often contain noise, outliers, or missing values, it may impair the analysis's accuracy."

- Replace the verb impair with compromise

>> The word “impair” has been replaced with “compromise” as suggested by reviewer.

- The following sentence is confusing in the description of EDA.

# “Exploratory Data Analysis (EDA) contains the methodology that includes statistical investigation...”

- Suggestion: Exploratory Data Analysis (EDA) includes methodologies such as statistical investigation, treatment of null and duplicate values, and identification of outliers...

>> The correction for EDA has been incorporated and highlighted as per the reviewer suggestion.

- The following sentence is poorly worded

# “Applying EDA is basic & essential process which considers 70% of work completion.”

- Suggestion: Performing EDA is fundamental and comprises approximately 70% of the total effort in the analysis process.

>> As per reviewers’ suggestion the correction has been incorporated and highlighted.

- The following explanation about when to use the Z and T-tests seems incorrect.

# “When the sample size is greater than or equal to 30, the Z-test is employed. T-Test uses unpaired and paired approach but the sample size should be below 30.”

- Suggestion: The Z-test is used when the population standard deviation is known, or the sample size is large (n ≥ 30). The T-test is used when the population standard deviation is unknown, and the sample size is small (n < 30).

>> The correction in Hypothesis testing section has been made and highlighted as per the reviewer suggestion.

- The following sentence is not precise about the use of tests in relation to standard deviation

# "Also, if the SD of data is known then it is preferred to use Z-test if it is not known then use T-test but still the sample size should be less than 30."

- Suggestion: If the population standard deviation is known, use the Z-test regardless of the sample size. If it is unknown and the sample is small, use the T-test.

>> The suggested correction has been incorporated and highlighted in hypothesis section.

- There is a lack of clarity in the following sentence

# "Z-test has failed to reject the null hypothesis... Anova test has also fail to reject Null Hypothesis..."

- Suggestion: The Z-test failed to reject the null hypothesis, indicating no significant difference between the means of the training and test sets for the “casing” feature. Likewise, the ANOVA test failed to reject the null hypothesis, suggesting that the means of the original, training, and test sets are statistically equal.

>> The suggested correction has been incorporated and highlighted in hypothesis section.

- The caption for Figure 5 has some typographical issues

# “Figure5:HypothesisTestingofCPMdataset”

>> The typographical correction has been incorporated and highlighted in the figure of hypothesis testing as suggested by reviewer.

- The hypothesis testing section uses complex language that can be simplified.

# "The p-value has to be determined for sample data on the basis of that null and alternate hypothesis test will give the outcomes for z-test & anova test. So, applying hypothesis for stats comparison of CPM data before and after preprocessing. The z-test is used to determine if there is a significant difference in the means of the features, whereas ANOVA is applied to check the variance across multiple groups, allowing us to ensure data consistency and quality."

- Simplify the language and explanation of the tests, focusing on the main conclusions.

>> As per reviewers’ suggestion the entire Hypothesis section has been simplified and the conclusive part has been added.

Result and discussion

- A superficial description of the univariate analyses was provided.

# "Univariate analysis, which examines one variable at a time, has been employed..."

- Provide specific details of the analyses performed and their results. This may help explain why there are 03 different graphs for the same variables, as discussed below.
- In the subsections Histogram Plot and Violin Analysis, Box Plot analysis
  - There is no mention of which variable is being measured, the frequency. For example, is the rotation or vibration of the Casing, Impeller, and Bearing being measured?
  - Why use 03 different graphs to present the same variable? I suggest choosing and keeping either a histogram, violin, or box plot.

>> As per reviewers’ suggestion the histogram and violin plots were removed and only one plot “box plot” has been included because of same variable information.

- The following sentence is poorly formulated about Naive Bayes
  - "It's commonly used for Multi Classification."
  - Correct to: "It's commonly used for multiclass classification problems."

>> The suggested correction has been incorporated and highlighted in Naïve bayes section.

Conclusion:

- Inadequate insertion of a table in conclusion.
  - Table 4 Performance Table
  - Should be in the results and discussion section
  - The caption of the Table should be more specific about the performance of what is being presented on the table. Change the name of the caption to be more complete about what the table presents.

>> The entire table 4 has been shifted in Results & discussion section as suggested by reviewer. Also, the name of table has been changed and highlighted in the section.

- Contradiction about the performance of the RF model.

# "However, the Random Forest classifier showed tendencies to overfit..."

- Explain in the results section, justifying the statement about Random Forest overfitting.

>> The explanation has been incorporated in the Random Forest classifier section and conclusion as per the reviewer suggestion.

For all sections:

- Check the quality of the Figures
  - For example, violin plots and box plots have low resolution and are difficult to interpret.
  - Improve the resolution of the figures by exporting them to high-quality formats (for example, PNG with 300 dpi).
  - Standardize the style of the graphs, using axes with captions and clearly indicated units

>> As suggested by reviewer and taking reference of mail by Plos one all the images of the manuscript have been converted to .tiff file format and 300 dpi quality.
