# Supplementary material for: Innovative data techniques for centrifugal pump optimization with machine learning and AI model
Source: PLoS One. 2025 Jun 10;20(6):e0325952. doi: 10.1371/journal.pone.0325952 (PMC12151477; doi:10.1371/journal.pone.0325952)
Supplement: Attachment — Submitted filename: Response_to_Reviewers_auresp_2.docx [file pone.0325952.s004.docx]

**Review Comments to the Author**

Reviewer Comment #4: Major revision required to improve clarity, methodological rigor, and language. The authors should address inconsistencies in statistical interpretations, ensure ML model transparency, and refine figures/tables for readability.

>> All the changes has been incorporated as per reviewer’s suggestion.

• The abstract presents a broad summary but does not include numerical results. Sentences like "this approach improves operational efficiency" need quantification (e.g., percentage increase in efficiency, reduction in downtime).

>> As per the reviewer’s suggestion the percentage increase on efficiency and reduction in model training and downtime details has been incorporated in abstract.

• The phrase "the quality of data recorded plays the important role" is grammatically incorrect and should be revised for readability. Additionally, "machine learning (ML) & Deep Learning models (DL)" should be consistently formatted throughout the paper.

>> The correction in abstract has been incorporated as per reviewers’ suggestion

• Weak Problem Statement. The introduction mentions challenges in centrifugal pump monitoring but does not specify why current machine learning techniques are insufficient. The research problem should clearly define the existing gaps in literature with specific citations.

>> The correction as suggested by reviewer has been incorporated with specific citation in 2^nd^ last paragraph of introduction.

• Lack of Theoretical Background. The study mentions the application of ML and AI but does not provide a theoretical framework linking these methods to predictive maintenance or anomaly detection in pump systems. Referencing established theories in condition monitoring and fault detection would strengthen the argument.

>> As per the reviewer’s suggestion the theoretical background of linking the predictive maintenance and aiml has been added in first paragraph of introduction.

• The same references appear multiple times without adding new insights. The literature review should be more structured, categorizing studies into specific themes (e.g., feature engineering, predictive maintenance, deep learning architectures).

>> As per reviewer’s suggestion some of the repeated reference has been removed after analysing. The new insights have been added for some of the repeated references that were necessary to cite again.
• The paper mentions feature engineering techniques but does not explain why specific feature selection methods (e.g., PCA, mutual information) were chosen over others. A justification for the methodology is needed.

>> The correction has been incorporated and highlighted as per reviewer’s suggestion.

• Some papers are important to cite in the literature.
1. Active learning-based machine learning approach for enhancing environmental sustainability in green building energy consumption
2. Integrating machine and deep learning technologies in green buildings for enhanced energy efficiency and environmental sustainability

>> The paper has been beneficial for understanding the optimal data preprocessing, EDA , ML workflow and cited as per reviewers’ suggestion

• Inadequate justification of data selection and sample size.
• The dataset consists of 70,062 rows and 13 columns, but the paper does not explain why this data size is appropriate for training ML models. Was a power analysis or cross-validation strategy applied to confirm adequacy?

>> This research is continuation of my previous work; and as now it is published, I have cited the research paper where I have experimentally performed and statistically selected the authenticated DAQ system as well as data. The correction has been incorporated in “basic architecture flow of model” as per reviewer’s suggestion.

• The study mentions data cleaning and transformation, but does not describe how missing values, outliers, or imbalanced classes were handled. These preprocessing steps are crucial for ML performance and should be explicitly detailed.

>> The Synthetic Minority Over-Sampling Technique (SMOTE) from imblearn library was used to increase minority class samples and achieve balance. Also, no missing values were found as it was been taken care while data preparation. The changes have been incorporated and highlighted as per reviewer’s suggestion

• While the paper presents means and standard deviations, there is no explanation of what these values imply in the context of pump optimization. Does higher variance in sensor data indicate potential faults?

>> Yes, Higher Variance in sensor data from the mean indicate potential faults. The correction has been incorporated and highlighted as per reviewer’s suggestion.

• **The regression analysis claims that sensor temperature has a strong correlation with performance, but this contradicts previous claims that flow rate is the primary predictor**

>> The heat maps indicate a strong correlation between DC_RA and B_temp, reflecting that increased cavitation corresponds with elevated bearing temperature. Bearing temperature serves as a predictor for cavitation in both casing of impeller and impeller. In a Good Health Condition (GHC), the flow rate exhibits a positive correlation with pressure. However, 75% of the data pertains to faulty conditions, wherein MA significantly reduces flow. Consequently, the heat map reveals a negative correlation that adversely impacts pump performance.

As per reviewer’s suggestion, the correction has been integrated into the heatmap analysis and highlighted, thereby enhancing the strong data's interpretive insights.

• The study reports high ML accuracy (99.99%), but does not compare against simpler baseline models (e.g., logistic regression). Without a benchmark comparison, the claims about model superiority are unconvincing.

>>As per reviewer’s suggestion, The logistic model has been incorporated with its performance and highlighted.

• The deep learning model achieved 100% accuracy on both training and testing data, which suggests severe overfitting. Did the authors apply regularization techniques (dropout, L2 penalty) to mitigate overfitting?

>> As per reviewer’s suggestion the deep learning model was trained with regularizing technique: The dropout rate with 0.3 and L2 regularization with 0.001 was performed. The correction has been incorporated and highlighted in the manuscript.

• The conclusion overstates the success of ML models without acknowledging limitations, such as potential biases in sensor data, generalizability to different pump types, and interpretability challenges.

>> The limitation has been incorporated in the conclusion as per reviewer’s suggestion

• Significant grammatical errors and formatting inconsistencies.

>> As per suggestion by reviewer the correction has been incorporated & highlighted.
